# Sensorless Speed Control of PMSMs Based on an Improved Fast Power Reaching Law

**DOI:** 10.3390/s26123737

**Published:** 2026-06-11

**Authors:** En Lu, Yufei Liu, Minghui Zhang, Jinyong Ju

**Affiliations:** 1 Jiangsu Engineering Research Center of Key Technology for Intelligent Manufacturing Equipment, Suqian University, No. 399 Huanghe South Road, Suqian 223800, China; 2 School of Agricultural Engineering, Jiangsu University, No. 301 Xuefu Road, Zhenjiang 212013, China; 3 School of Artificial Intelligence, Anhui Polytechnic University, No. 8 Beijing Middle Road, Wuhu 241000, China; 4 Weichai Lovol Intelligent Agricultural Technology Co., Ltd., No. 192 Beihai Road, Weifang 261000, China

**Keywords:** PMSM, sensorless control, improved fast power reaching law, non-singular terminal sliding mode, sliding mode observer

## Abstract

Traditional permanent magnet synchronous motor (PMSM) control systems rely on mechanical position sensors for high-precision rotor position and speed information, which increases hardware complexity, raises system cost, reduces reliability, and limits adaptability to harsh environments. To overcome the above limitations, this paper proposes a novel high-performance sensorless speed control strategy for PMSMs, which is constructed based on a non-singular terminal sliding mode observer (NTSMO) and a non-singular terminal sliding mode controller (NTSMC). First, an improved fast power reaching law (IFPRL) is proposed, which consists of a variable exponential reaching term and a power reaching term. Specifically, the gain of the exponential reaching term is dynamically adjusted by the absolute value of the sliding mode switching function, enabling the reaching law to operate in two different modes throughout the entire convergence process of the system state. Moreover, the introduction of scaling coefficient *c* compensates for the performance degradation caused by variations in the range of sliding mode surfaces (SMSs) in different systems. The proposed IFPRL not only effectively mitigates the inherent chattering issue, it also expedites the rate at which the system state converges to its SMS. On this basis, both the NTSMO for rotor position observation and the NTSMC for speed closed-loop control are designed by embedding the proposed IFPRL into the framework of non-singular terminal sliding mode control theory. Finally, the effectiveness of the proposed method is validated through numerical simulations and experimental tests. Experimental results demonstrate that the proposed IFPRL-based NTSMC + NTSMO scheme reduces the root mean square error (RMSE) of speed control by 2.7% relative to the traditional SMC + SMO method. The proposed method realizes reliable sensorless speed control for PMSMs and exhibits superior dynamic response, higher control accuracy, and stronger robustness against disturbances.

## 1. Introduction

Owing to their distinct advantages, including a simple structure, high operating efficiency, and high-power density, permanent magnet synchronous motors (PMSMs) have been widely adopted in a broad range of applications such as aerospace, computer numerical control machine tools, new energy vehicles, mining machinery, and other industrial scenarios [1,2]. The high-performance control of PMSMs relies critically on high-precision rotor position information, which is conventionally obtained through physical sensors such as photoelectric encoders and resolvers. Nevertheless, the deployment of such sensors introduces additional electronic circuitry, regular preventive maintenance, and extra wiring harnesses. Furthermore, under harsh operating conditions characterized by severe vibration and mechanical shock, these position sensors are highly prone to failure, which directly undermines the long-term reliability and environmental robustness of the entire PMSM drive system. Consequently, the elevated system cost and compromised reliability associated with mechanical position sensors have motivated extensive research into sensorless speed control strategies for PMSMs [3].

At present, sensorless speed control schemes for PMSMs can generally be divided into methods based on the motor fundamental wave model and methods based on salient pole tracking, mainly including the high-frequency signal injection method [4], the flux estimation method [5], the model reference adaptive method [6], and the counter-electromotive force observation method [7,8,9], etc. Among them, the counter-electromotive force observation method, as represented by the sliding mode observer (SMO), is the most extensively utilized approach.

The core principle of SMO is rooted in sliding mode variable structure control theory, where the real-time estimation error is treated as a lumped system disturbance, and the control output is dynamically adjusted via a switching function to drive the system states to converge along a predefined sliding mode surface (SMS) [10]. The SMS of the system can be independently designed and is inherently insensitive to internal parameter perturbations and external load disturbances. Therefore, strong robustness and a low dependence on precise motor model parameters are the most prominent advantages of SMOs [11]. Meanwhile, the design of a speed controller using sliding mode control (SMC) theory can significantly enhance both the steady-state speed tracking accuracy and anti-disturbance performance of PMSM drive systems under complex and variable operating conditions.

However, keeping the system states strictly on the SMS remains a key technical challenge, arising from the inherent discontinuous switching characteristics of SMC and the inertia constraints of the physical actuator. These drawbacks typically lead to slow convergence rates and severe chattering in the control output. To suppress the inherent chattering problem, some researchers have replaced the discontinuous switching function with a continuous sigmoid function for SMO design [12,13]. Yang et al. proposed an improved switching function with a dual-boundary-layer structure [14]. However, these methods tend to degrade the steady-state estimation accuracy of state variables under system disturbances and parameter uncertainties. Furthermore, the adoption of soft-switching functions instead of conventional hard-switching functions will slow down the system’s convergence speed to the sliding mode surface (SMS) and reduce the rotor position estimation accuracy.

The chattering phenomenon is caused by the system trajectory’s defective reach process towards the SMS, which is ultimately determined by the quality of the reaching law. Therefore, exploring high-quality reaching law is the key to suppressing system chattering [15]. Chen et al. proposed an adaptive fixed-time integral sliding mode controller based on fixed-time stability theory, which ensures that the system settling time is independent of initial conditions and that the speed tracking error converges within a finite time [16]. Wang et al. proposed a novel sliding mode reaching law that introduces a power term associated with the system state variables, with the range of the power term limited by the absolute value of the switching function [17]. Cheng et al. proposed a fractional-order SMS and reaching law to increase the convergence speed and suppress the chattering present in traditional SMOs [18]. Tang realized chattering-free sliding mode motion through the matching design of the NFTSM sliding surface and the NRL, fundamentally suppressed the inherent chattering defect of traditional SMCs, and greatly optimized the dynamic response performance of the system [19].

In order to further improve the dynamic performance of the sensorless speed control system for PMSMs, this paper proposes a novel improved fast power reaching law (IFPRL), which integrates a variable exponential reaching term and a power reaching term. This enables the reaching law to take two distinct mathematical forms when the exponential reaching term is bounded by the absolute value of the switching function within a unit magnitude (|·| ≤ 1). This design not only accelerates the convergence speed of the system state to the sliding mode surface, it also ensures smooth convergence of the system state to the switching surface. On this basis, the rotor position observer and speed controller for PMSMs are designed separately to realize sensorless speed control of PMSMs with superior dynamic performance and strong robustness. The following is a summary of this work’s innovations and contributions:

(a) A novel IFPRL incorporating a power reaching term and a variable exponential reaching term was proposed. Notably, the exponential reaching term is limited by the absolute value of the switching function. This allows the designed IFPRL to have two different representations during the arrival process. Furthermore, the introduction of an adjustable coefficient *c* addresses the problem of decreased reaching law performance caused by different ranges of SMSs in different systems.
(b) According to the designed IFPRL and non-singular terminal SMC theory, this paper constructs an IFPRL-based NTSMO and realizes the rotor position estimation for the PMSM by combining PLL technology. Similarly, based on the proposed IFPRL, an IFPRL-based non-singular terminal sliding mode controller (NTSMC) was designed for the PMSM speed control. Finally, the effectiveness of the proposed sensorless speed control method was verified through numerical simulations and experiments.

The structure of this paper is outlined as follows: The mathematical modeling is explained in Section 2. Section 3 introduces the design method of IFPRL. Section 4 designs a rotor position observer based on an IFPRL. Section 5 designs a speed controller based on the IFPRL and provides the sensorless speed control framework. Section 6 gives the outcomes of numerical simulations and experiments. Section 7 provides the brief conclusions.

## 2. Mathematical Model of PMSM

**Figure 1:**
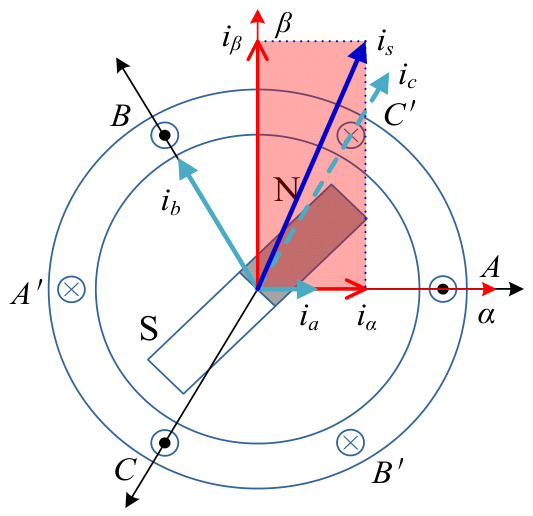
Clarke transformation.

According to the standard coordinate transformation theory applied in AC motor drive systems [20], the three-phase stator currents of a PMSM can be transformed into their equivalent two-phase counterparts in the stationary α-β reference frame through the Clarke transformation [2]. This paper focuses on a surface-mounted PMSM. As shown in Figure 1, the resultant current vector formed by the three-phase stator currents (*i_a_*, *i_b_* and *i_c_*) can be decomposed into two mutually perpendicular components. In this decomposition, the *α*-axis of the two-phase stationary coordinate system is aligned with the A-axis of the three-phase stationary coordinate system, while the *β*-axis is counterclockwise ahead of the *α*-axis at a 90° spatial electrical angle. The Clarke transformation is presented as follows:

(1) $$\left[\begin{array}{c} i_{\alpha }\\ i_{\beta } \end{array}\right]=\frac{2}{3}\left[\begin{array}{ccc} 1 & \cos\left(\frac{2\pi }{3}\right) & \cos\left(\frac{4\pi }{3}\right)\\ 0 & \sin\left(\frac{2\pi }{3}\right) & \sin\left(\frac{4\pi }{3}\right) \end{array}\right]\left[\begin{array}{c} i_{a}\\ i_{b}\\ i_{c} \end{array}\right]$$

where *i_a_*, *i_b_* and *i_c_* represent the PMSM’s three-phase currents, and *i_α_* and *i_β_* represent the respective currents along the *α*-axis and *β*-axis.

Therefore, the PMSM’s voltage equations in the *α*-*β* coordinate system are presented as follows:

(2) $$\left\{\begin{array}{c} u_{\alpha }=(R_{s}+p_{n}L_{d})i_{\alpha }+\omega_{e}(L_{d}-L_{q})i_{\beta }+e_{\alpha }\;\;\;\;\;\;\;\;\;\;\;\\ u_{\beta }=-\omega_{e}(L_{d}-L_{q})i_{\alpha }+(R_{s}+p_{n}L_{q})i_{\beta }+e_{\beta }\;\;\;\; \end{array}\right.$$

where *R_s_* represents the stator resistance, *p_n_* represents the pole number of the PMSM, *ω_e_* represents the electrical angular speed (*ω_e_* = *p_n_ω_m_*, with *ω_m_* being the mechanical angular speed), *L_d_* and *L_q_* represent the respective inductances along the *d*-axis and *q*-axis, and *e_α_* and *e_β_* represent the counter-electromotive forces, respectively. In this case, the counter-electromotive forces *e_α_* and *e_β_* can be formulated as:

(3) $$\left[\begin{array}{c} e_{\alpha }\\ e_{\beta } \end{array}\right]=\left[(L_{d}-L_{q})(\omega_{e}i_{d}-p_{n}i_{q})+\omega_{e}\psi_{f}\right]\;\;\;\left[\begin{array}{c} -\sin\theta_{e}\\ \cos\theta_{e} \end{array}\right]$$

where *θ_e_* represents the position electrical angle, and *ψ_f_* represents the flux linkage.

**Figure 2:**
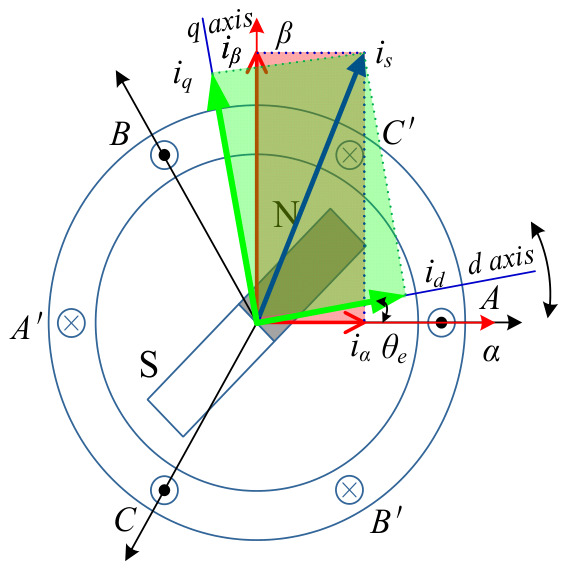
Park transformation.

The coordinate transformation from the two-phase stationary coordinate system to the synchronous rotating (d-q) coordinate system is referred to as the Park transformation [2]. As shown in Figure 2, the synchronous rotating coordinate system rotates in space at the same electrical angular velocity as the PMSM rotor. Specifically, the *d*-axis of the synchronous rotating coordinate system aligns with the rotor axis, while the *q*-axis is counterclockwise ahead of the *d*-axis at a 90° spatial electrical angle.

The Park transformation is presented as follows [21]:

(4) $$\left[\begin{array}{c} i_{d}\\ i_{q} \end{array}\right]=\left[\begin{array}{cc} \cos\theta_{e} & \sin\theta_{e}\\ -\sin\theta_{e} & \cos\theta_{e} \end{array}\right]\;\left[\begin{array}{c} i_{\alpha }\\ i_{\beta } \end{array}\right]$$

where *i_d_* and *i_q_* represent the stator current components along the *d*-axis and *q*-axis.

However, in sensorless control applications, the true rotor position *θ*_e_ is inherently unmeasurable. Instead, the estimated electrical rotor position ${\hat{\theta }}_{e}$ (output from the NTSMO designed in Section 4) is adopted to perform the Park transformation in practical implementation, yielding the stator current components (${\hat{i}}_{d}$, ${\hat{i}}_{q}$) in the estimated synchronous rotating ($\hat{d}-\hat{q}$).

The PMSM’s stator voltage equations in the synchronous rotating coordinate (*d*-*q*) are presented as follows [22]:

(5) $$\left\{\begin{array}{c} u_{d}=R_{s}i_{d}+L_{d}{\dot{i}}_{d}-\omega_{e}L_{q}i_{q}\;\;\;\;\;\;\;\;\;\;\;\;\;\;\;\;\;\;\;\;\;\;\;\;\;\;\;\;\;\;\;\\ u_{q}=R_{s}i_{q}+L_{q}{\dot{i}}_{q}+\omega_{e}L_{d}i_{d}+\omega_{e}\psi_{f} \end{array}\right.$$

The PMSM’s electromagnetic torque equation is presented as follows [23]:

(6) $$T_{e}=1.5p_{n}i_{q}[i_{d}(L_{d}-L_{q})+\psi_{f}]$$

where *p_n_* represents the count of pole pairs constituting the PMSM.

The PMSM’s motion equation is presented as follows:

(7) $$T_{e}-T_{L}=B_{m}\omega_{m}+J_{m}{\dot{\omega }}_{m}$$

where *T_Lm_* represents the PMSM’s load torque, *B_m_* represents the coefficient of viscous friction, and *ω_m_* represents the angular speed, respectively.

## 3. Design and Analysis of IFPRL

### 3.1. Design of IFPRL

In the SMC process, a well-designed reaching law is critical to guaranteeing the dynamic performance of the system during the reaching phase. Academician Gao first proposed the exponential reaching law (ERL) [24], which is given by:

(8) $$ds/dt=-k_{1}\mathrm{sgn}(s)-k_{2}s$$

where *k*_1_ and *k*_2_ represent positive real numbers, *k*_1_sgn(*s*) represents the isokinetic reaching term, and *k*_2_*s* represents the exponential reaching term. However, the conventional ERL [24] cannot guarantee finite-time convergence to the SMS. To ensure that the reaching speed is *k*_1_ rather than 0 when *s* is near to zero, the isokinetic reaching term *k*_1_sgn(*s*) must be introduced [25].

Assume that the system reaches the SMS for the first time at time *t*_1_, then the reaching time can be calculated by integrating Equation (8) from 0 to *t*_1_, and we can obtain [15]:

(9) $$t_{1}=1/k_{2}\ln\left[1+(k_{2}/k_{1})\left|s_{0}\right|\right]$$

where *s*_0_ represents the initial value of the SMS. Equation (9) indicates that the reaching speed rises as *k*_2_ increases. Hence, a larger *k*_2_ is preferred to achieve favorable reaching performance. However, when *s* reaches the SMS, a larger *k*_2_ may lead to too fast a speed of convergence, which will increase the chattering of the system [25].

This gives rise to an inherent dilemma: improving the reaching speed is inherently contradictory to suppressing sliding mode chattering [17]. To construct a dynamic mapping between the sliding mode reaching speed and the deviation of the system state from the SMS, a power function of the sliding variable is introduced into the exponential term. Meanwhile, the exponent of the exponential term is designed as a time-varying parameter to resolve the aforementioned contradiction between reaching speed and chattering suppression. By further integrating the power reaching law, the proposed IFPRL is shown as:

(10) $$ds/dt=-k_{1}{\left|s/c\right|}^{a}\mathrm{sgn}(s)-k_{2}{\left|s/c\right|}^{b\cdot \mathrm{sgn}(\left|s/c\right|-1)}s$$

where *k*_1_ > 0, *k*_2_ > 0, 0 < *a*<1, *b* > 0, and *c* > 0. The proposed IFPRL comprises two components: a power reaching term and a variable-parameter exponential reaching term. In addition, a scaling coefficient *c* is introduced to mitigate the performance degradation of the reaching law caused by the varying value ranges of the SMS in different systems.

In the scenario where the system state significantly deviates from the SMS, i.e., |*s*/*c*| > 1, then sgn(|*s*/*c*|−1) = 1, and the IFPRL shown in Equation (10) can be rewritten as *ds*/*dt* = −*k*_1_|*s*/*c*|*^a^*sgn(*s*) −*k*_2_|*s*/*c*|*^b^s*. At this point, |*s*/*c*| will gradually decrease under the action of the IFPRL. This implies that as the system state gradually approaches the SMS, the coefficients of both the power reaching term and the variable exponential reaching term will gradually decrease. This can have the effect of suppressing chattering. On the other side, when the system state approaches the SMS, i.e., |*s*/c| < 1, then sgn(|*s*/*c*|−1) = −1, and the IFPRL shown in Equation (10) can be rewritten as *ds*/*dt* = −*k*_1_|*s*/*c*|*^a^*sgn(*s*) −*k*_2_|*s*/*c*|^−*b*^*s*. At this point, obviously, there is *k*_2_|*s*/*c*|^−*b*^*s* >*k*_2_|*s*/*c*|*^b^s*, leading to an increased reaching speed of the variable exponential reaching term. Meanwhile, the small SMS at this time ensures that the control gain *k*_1_|*s*/*c*|*^a^* in the power reaching term is small, thereby reducing the system’s chattering.

### 3.2. Analysis of IFPRL

To conduct a comparative analysis between the designed IFPRL and other reaching laws, a typical motor rotation system depicted in Equation (11) as [25]:

(11) $$J\ddot{\theta }(t)=u(t)+d(t)$$

where *J* represents the moment of inertia, *θ*(*t*) represents the output angle, *u*(*t*) represents the control input, and *d*(*t*) represents the lumped disturbance.

Selecting the following SMS:

(12) $$s(t)=\varepsilon e(t)+\dot{e}(t)$$

where *ε* > 0 satisfies the Hurwitz condition, *θ_d_*(*t*) represents the reference angle, $e(t)=\theta (t)-\theta_{d}(t)$, and $\dot{e}(t)=\dot{\theta }(t)-{\dot{\theta }}_{d}(t)$.

The control input based on SMC is designed as:

(13) $$u=J(-\varepsilon \dot{e}+{\ddot{\theta }}_{d}-k_{1}{\left|s/c\right|}^{a}\mathrm{sgn}(s)-k_{2}{\left|s/c\right|}^{b\cdot \mathrm{sgn}(\left|s/c\right|-1)}s)$$

Using MATLAB/Simulink 2024b for simulation analysis, and selecting *J* = 10, *d* = sin(2*πt*), *T_s_* = 0.02 s, *θ*(0) = 0.5, and $\dot{\theta }(0)=1$. Select the traditional ERL [24], the reaching law in ref. [17] (NSMRL), and compare them with the IFPRL proposed in this paper. The parameters of the ERL are as follows: *ε* = 0.5, *k*_1_ = 0.5, and *k*_2_ = 1; the parameters of the NSMRL are as follows: *ε* = 0.5, *k*_1_ = 0.5, *k*_2_ = 1, *a* = 0.3, and *b* = 0.2; Tte parameters of the IFPRL are follows: *ε* = 0.5, *k*_1_ = 0.5, *k*_2_ = 1, *a* = 0.3, *b* = 0.2, *c* = 0.1.

**Figure 3:**
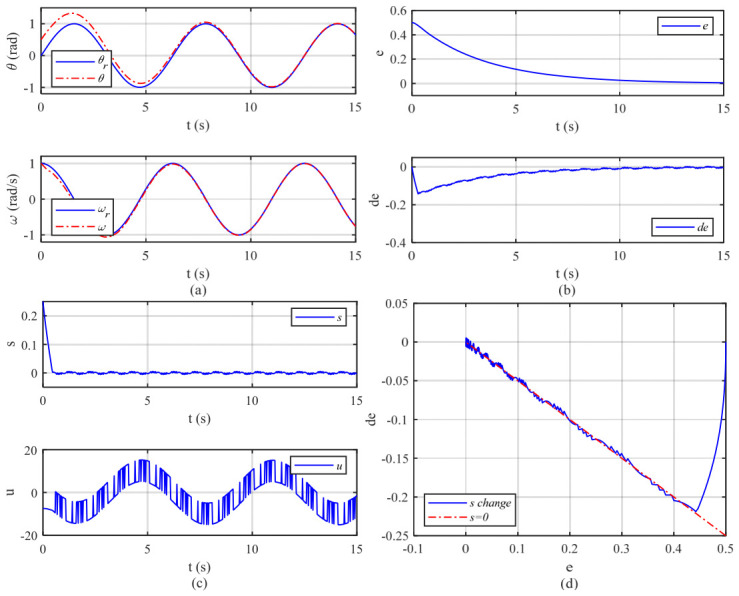
Control performance of the ERL: (**a**) position tracking; (**b**) tracking errors; (**c**) SMS and control input; (**d**) phase trajectory.

**Figure 4:**
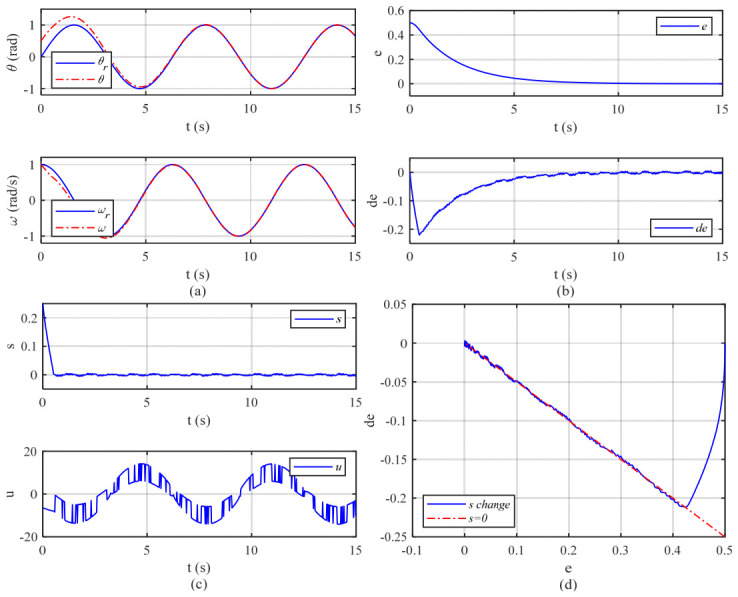
Control performance of the NSMRL: (**a**) position tracking; (**b**) tracking errors; (**c**) SMS and control input; (**d**) phase trajectory.

**Figure 5:**
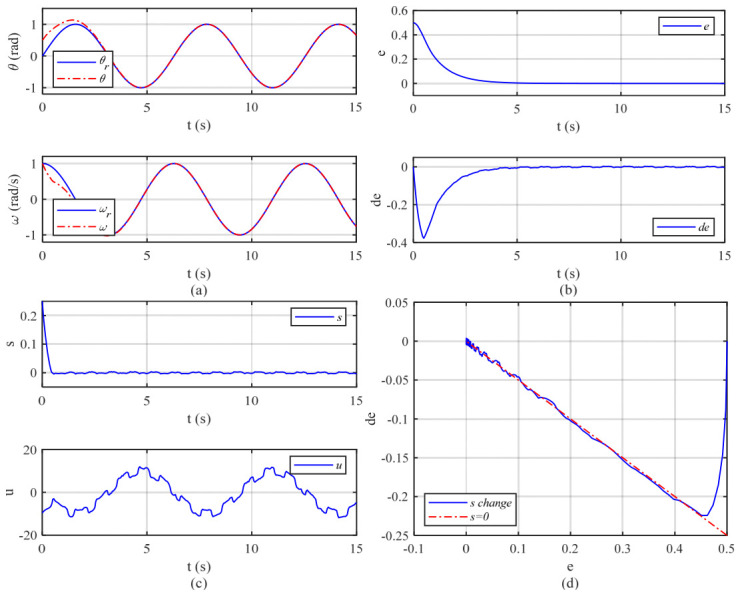
Control performance of the IFPRL: (**a**) position tracking; (**b**) tracking errors; (**c**) SMS and control input; (**d**) phase trajectory.

The simulation results are shown in Figure 3, Figure 4 and Figure 5, respectively. From the angle position tracking results shown in Figure 3a, Figure 4a and Figure 5a, it can be seen that all three reaching laws can effectively achieve tracking control. The tracking errors are shown in Figure 3b, Figure 4b and Figure 5b, and the system convergence accuracy is 0.0001047, 0.0000916, and 0.0000538, respectively. Figure 3c, Figure 4c and Figure 5c show the SMSs and control inputs of the three reaching laws. The chattering in Figure 3c is very obvious. The chattering in Figure 4c is also large, and the NSMRL [17] did not have the expected effect of reducing chattering. This is because the SMS *s* has always been less than 1, so the variable exponential term in the NSMRL [17] cannot play the role of variable index. The chattering is basically suppressed in Figure 5c. This is because the coefficient *c* is introduced into the IFPRL, which ensures that the variable exponential term in the IFPRL can play the expected role when the SMS *s* is small. The phase trajectories of sliding mode motion are shown in Figure 3d, Figure 4d and Figure 5d. In conclusion, the IFPRL proposed in this paper is superior to ERL [24] and NSMRL [17] in tracking the given signal, reducing the position tracking error, improving the convergence rate of tracking error, and suppressing chattering. Therefore, the IFPRL proposed in this paper effectively solves the conflict between chattering suppression and fast response, and has the advantage of a more stable controller output effect.

## 4. Design of Rotor Position Observer Based on IFPRL

### 4.1. Design of NTSMO

This paper designs the NTSMO based on the synchronous rotating coordinate system, considering *i_d_*, *i_q_*, and *ω_e_* as the state variables in the system. In practical implementation, the three-phase stator currents *i_a_*, *i_b_*, and *i*_c_ are directly measured [26], and then transformed into the *d*-*q* coordinate system using the estimated rotor angle ${\hat{\theta }}_{e}$ to obtain the feedback currents ${\hat{i}}_{d}$ and ${\hat{i}}_{q}$ [27,28]. The control inputs *u_d_* and *u_q_* are generated by the controller and then transformed back to the three-phase stationary coordinate system using ${\hat{\theta }}_{e}$ to drive the inverter. According to Equation (5), the rewritten stator current equation is as follows:

(14) $$\left\{\begin{array}{c} {\dot{i}}_{d}=\left(-R_{s}i_{d}+L_{q}\omega_{e}i_{q}+u_{d}-E_{d}\right)/L_{d}\;\;\;\\ {\dot{i}}_{q}=\left(-R_{s}i_{q}-\omega_{e}L_{d}i_{d}+u_{q}-E_{q}\right)/L_{q}\;\;\;\; \end{array}\right.$$

where $E_{d}=0$, $E_{q}=\omega_{e}\psi_{f}$ represent the extended counter-electromotive forces in the *d*-*q* axes. *E_q_* contains rotor speed information; thus, accurate estimation of *E_q_* enables the acquisition of rotor speed and position. The SMO is constructed as follows:

(15) $$\left\{\begin{array}{c} {\dot{\hat{i}}}_{d}=\left(-R_{s}{\hat{i}}_{d}+L_{q}{\hat{\omega }}_{e}{\hat{i}}_{q}+u_{d}-V_{d}\right)\;/L_{d}\;\;\;\;\;\;\;\;\;\;\;\;\;\;\;\;\;\;\;\\ {\dot{\hat{i}}}_{q}=\left(-R_{s}{\hat{i}}_{q}-{\hat{\omega }}_{e}L_{d}{\hat{i}}_{d}+u_{q}-V_{q}-{\hat{E}}_{q}\right)/L_{q} \end{array}\right.$$

where ${\hat{i}}_{d}$ and ${\hat{i}}_{q}$ are the estimated values of the stator currents; ${\hat{\omega }}_{e}$ is the estimated value of the electrical angular speed *ω_e_*; ${\hat{E}}_{q}$ is the estimated value of the counter-electromotive force *E_q_*; and *V_d_* and *V_q_* are the SMC laws to be designed.

Define the current estimation errors as ${\tilde{i}}_{d}={\hat{i}}_{d}-i_{d}$, ${\tilde{i}}_{q}={\hat{i}}_{q}-i_{q}$. Subtracting Equation (14) from Equation (15) yields the error dynamics:

(16) $$\left\{\begin{array}{c} {\dot{\tilde{i}}}_{d}=\left(-R_{s}{\tilde{i}}_{d}+L_{q}(\omega_{e}{\hat{i}}_{q}-{\hat{\omega }}_{e}{\hat{i}}_{q})-V_{d}\right)/L_{d}\;\;\;\;\;\;\;\;\;\;\;\;\;\;\;\;\;\;\;\\ {\dot{\tilde{i}}}_{q}=\left(-R_{s}{\tilde{i}}_{q}-L_{d}(\omega_{e}{\hat{i}}_{d}-{\hat{\omega }}_{e}{\hat{i}}_{d})-V_{q}-{\tilde{E}}_{q}\right)/L_{q} \end{array}\right.$$

where ${\tilde{E}}_{q}={\hat{E}}_{q}-E_{q}$ is the estimation error of the counter-electromotive force.

The aforementioned state equation of current observation errors can be reformulated into a matrix form [8]:

(17) $$\dot{\tilde{i}}=A\tilde{i}+BV+D$$

where $\tilde{i}={\left[{\tilde{i}}_{d},\;\;\;{\tilde{i}}_{q}\right]}^{T}$, $A=\left[\begin{array}{cc} -R_{s}/L_{d} & L_{q}\omega_{e}/L_{d}\\ -L_{d}\omega_{e}/L_{q} & -R_{s}/L_{q} \end{array}\right]$, $B=\left[\begin{array}{cc} 1/L_{d} & 0\\ 0 & 1/L_{q} \end{array}\right]$, $V={\left[V_{d},\;\;\;V_{q}\right]}^{T}$ is SMC law, and $D={\left[\frac{L_{q}}{L_{d}}(\omega_{e}i_{q}-{\hat{\omega }}_{e}{\hat{i}}_{q}),\;\;\;-\frac{L_{d}}{L_{q}}(\omega_{e}i_{d}-{\hat{\omega }}_{e}{\hat{i}}_{d})-\frac{{\tilde{E}}_{q}}{L_{q}}\right]}^{T}$ is the lumped disturbance vector, which comprehensively includes the effects of speed estimation error, counter-electromotive force estimation error, and parameter perturbations.

Considering taking $\tilde{i}$ as the state variable, the non-singular SMS is designed as follows [25]:

(18) $$s_{o}=\tilde{i}+\beta^{-1}{\dot{\tilde{i}}}^{p/q}$$

where ***s****_o_* = [*s_d_*, *s_q_*]*^T^*, ${\dot{\tilde{i}}}^{p/q}={\left[{\dot{\tilde{i}}}_{d}^{p/q},\;\;\;{\dot{\tilde{i}}}_{q}^{p/q}\right]}^{T}$; *p*, *q* and ***β*** are design parameters; *p* and *q* are positive odd numbers (*p* > *q*); and ***β*** = diag{*β_d_*, *β_q_*}, *β_d_*, *β_q_* > 0. If ***s****_o_* converges to zero, $\tilde{i}$ and $\dot{\tilde{i}}$ will also converge to zero in a finite time. At this point, the system remains on the second-order sliding mode $\tilde{i}=\dot{\tilde{i}}=0$.

Taking the derivative of the non-singular SMS ***s****_o_* with respect to time, we obtain the following expression:

(19) $${\dot{s}}_{o}=\dot{\tilde{i}}+\frac{p}{q}\beta^{-1}{\dot{\tilde{i}}}^{p/q-1}\ddot{\tilde{i}}$$

For the stator current error equation shown in Equation (17), based on the SMS shown in Equation (18) and the IFPRL shown in Equation (10), we obtain the following control law [2,29]:

(20) $$V=V_{eq}+V_{n}$$

where $V_{n}=-\int_{0}^{t}\left[diag(L_{d},\;\;\;L_{q})\frac{\beta q}{p}{\dot{\tilde{i}}}^{2-p/q}+k_{1}{\left|s_{o}/c\right|}^{a}\mathrm{sgn}(s_{o})+k_{2}{\left|s_{o}/c\right|}^{b\cdot \mathrm{sgn}(\left|s_{o}/c\right|-1)}s_{o}+\dot{D}\right]d\tau$, $V_{eq}=-A^{’}\tilde{i}$, $A^{’}=\left[\begin{array}{cc} -R_{s} & L_{q}\omega_{e}\\ L_{d}\omega_{e} & -R_{s} \end{array}\right]$, and $\dot{D}$ denotes the derivative of the lumped disturbance vector. Due to the inherent strong robustness of SMC against disturbances, $\dot{D}$ can be regarded as a bounded quantity in practical applications, and its influence will be fully compensated by the SMC term.

### 4.2. Stability Analysis of NTSMO

Considering the following Lyapunov function:

(21) $$V_{o}=\frac{1}{2}s_{o}^{2}$$

By taking the time derivative of the Lyapunov function ***V****_o_*, we obtain the following expression:

(22) $$\begin{array}{l} \;\;\;\;\;{\dot{V}}_{o}=s_{o}\cdot {\dot{s}}_{o}=s_{o}\cdot \left(\dot{\tilde{i}}+\frac{p}{q}\beta^{-1}{\dot{\tilde{i}}}^{p/q-1}\ddot{\tilde{i}}\right)\\ \;\;\;\;\;\;\;\;\;\;=s_{o}\cdot \frac{p}{q}\beta^{-1}diag\{{\dot{\tilde{i}}}^{p/q-1}\}\left(\ddot{\tilde{i}}+\frac{\beta q}{p}{\dot{\tilde{i}}}^{2-p/q}\right) \end{array}$$

Expanding the observation error equation shown in Equation (17) and substituting Equation (20) into it can obtain:

(23) $$\dot{\tilde{i}}=BV_{n}+D$$

Thus, the expression for the second derivative of the observation error $\tilde{i}$ is as follows:

(24) $$\ddot{\tilde{i}}=B{\dot{V}}_{n}+\dot{D}$$

Substituting Equation (24) and Equation (20) into Equation (22), we can obtain:

(25) $$\begin{array}{l} \;\;\;\;\;\;\;\;\;{\dot{V}}_{o}=s_{o}\cdot \frac{p}{q}\beta^{-1}diag\{{\dot{\tilde{i}}}^{p/q-1}\}\left(-k_{1}{\left|s_{o}/c\right|}^{a}\mathrm{sgn}(s_{o})-k_{2}{\left|s_{o}/c\right|}^{b\cdot \mathrm{sgn}(\left|s_{o}/c\right|-1)}s_{o}\right)\\ \;\;\;\;\;\;\;\;\;\;\;\;\;\;=\frac{p}{q}\beta^{-1}diag\{{\dot{\tilde{i}}}^{p/q-1}\}\left(-ck_{1}{\left|s_{o}/c\right|}^{1+a}-k_{2}{\left|s_{o}/c\right|}^{b\cdot \mathrm{sgn}(\left|s_{o}/c\right|-1)}s_{o}^{2}\right) \end{array}$$

Due to *c* > 0, *k*_1_ > 0, *k*_2_ > 0, *p* and *q* (*p* > *q*) being positive odd numbers, the Equation (25) can be simplified as:

(26) $${\dot{V}}_{o}=\frac{p}{q}\beta^{-1}diag\{{\dot{\tilde{i}}}^{p/q-1}\}\left(-ck_{1}{\left|s_{o}/c\right|}^{1+a}-k_{2}{\left|s_{o}/c\right|}^{b\cdot \mathrm{sgn}(\left|s_{o}/c\right|-1)}s_{o}^{2}\right)\;\leq 0$$

Therefore, the NTSMO designed in this paper is stable.

### 4.3. Design of PLL

The aforementioned NTSMO is capable of estimating the induced counter-electromotive forces (*E_d_*, *E_q_*) along the *d*-*q* axes in the PMSM system. It can be seen from Equation (14) that the induced counter-electromotive force of the *q*-axis encompasses rotor speed information. Therefore, the rotor electrical angular speed of the PMSM can be calculated using the following formula [30]:

(27) $${\hat{\omega }}_{e}={\hat{E}}_{q}/\psi_{f}$$

However, the direct division method is highly sensitive to measurement noise and parameter variations, and is prone to numerical fluctuations when ${\hat{E}}_{q}$ crosses zero. To address these issues, this paper adopts the PLL technique to achieve robust rotor position estimation through closed-loop tracking of the counter-electromotive force phase. In the three-phase stationary coordinate system, the stator terminal voltages of the PMSM can be expressed as symmetric sinusoidal waveforms:

(28) $$\left\{\begin{array}{c} u_{a}=U\cos{\hat{\omega }}_{e}t\;\;\;\;\;\;\;\;\;\;\;\;\;\;\;\;\;\;\;\;\;\;\;\;\;\;\;\;\;\;\;\;\;\;\;\;\;\\ u_{b}=U\cos({\hat{\omega }}_{e}t-2\pi /3)\\ u_{c}=U\cos({\hat{\omega }}_{e}t+2\pi /3) \end{array}\right.$$

where *U* represents the terminal voltage amplitude. When transforming these voltages to the synchronous rotating (*d*-*q*) coordinate system using the Park transformation, the transformation matrix is given by [8]:

(29) $$T({\hat{\theta }}_{e})=\frac{1}{3}\left[\begin{array}{ccc} \cos{\hat{\theta }}_{e} & \cos\left({\hat{\theta }}_{e}-\frac{2}{3}\pi \right) & \cos\left({\hat{\theta }}_{e}+\frac{2}{3}\pi \right)\\ -\sin{\hat{\theta }}_{e} & -\sin\left({\hat{\theta }}_{e}-\frac{2}{3}\pi \right) & -\sin\left({\hat{\theta }}_{e}+\frac{2}{3}\pi \right) \end{array}\right]$$

where ${\hat{\theta }}_{e}$ represents the estimated phase angle, and ${\hat{\theta }}_{e}={\hat{\omega }}_{e}t$.

Substituting the transformation matrix shown in Equation (29) into Equation (28), considering that it generally lacks zero sequence components, it can be obtained that:

(30) $$\left[\begin{array}{c} {\hat{u}}_{d}\\ {\hat{u}}_{q} \end{array}\right]=\left[\begin{array}{c} U\sin({\hat{\theta }}_{e}-\theta_{e})\\ U\cos({\hat{\theta }}_{e}-\theta_{e}) \end{array}\right]$$

**Figure 6:**
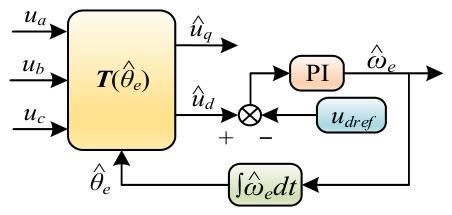
Schematic depiction of PLL.

In accordance with the definition of the synchronous rotating coordinate system, it is deduced that *u_dref_* = *u_d_* = 0. Consequently, a closed-loop PI regulator is designed utilizing Equation (30) [31], and the rotor position estimation principle based on PLL is shown in Figure 6.

Based on the expected bandwidth *ω_n_* of the closed-loop system, the parameters of the PI regulator in the PLL can be obtained as follows:

(31) $$\left\{\begin{array}{c} k_{p}=\sqrt{2}\omega_{n}/{\hat{u}}_{q}\\ k_{i}=\omega_{n}^{2}/{\hat{u}}_{q}\;\;\;\;\;\;\;\;\;\;\; \end{array}\right.$$

## 5. Design of Speed Controller Based on IFPRL

### 5.1. Design of NTSMC

Therefore, for the surface-mounted PMSM, selecting the rotor flux orientation control (*i_d_* = 0) scheme can achieve superior performance. According to Equations (5)–(7) and ignoring the effects of damping *B_m_*, the mathematical model of the linear steady-state system of the PMSM in the *d*-*q* coordinate system is shown as follows:

(32) $$\left\{\begin{array}{c} u_{d}=R_{s}i_{d}+L_{d}\frac{di_{d}}{dt}-p_{n}\omega_{m}L_{q}i_{q}\;\;\;\;\;\;\;\;\;\;\;\;\;\;\;\;\;\;\;\;\;\;\;\;\;\;\;\;\;\;\;\;\;\;\;\;\;\;\;\;\;\;\;\\ u_{q}=R_{s}i_{q}+L_{q}\frac{di_{q}}{dt}+p_{n}\omega_{m}L_{d}i_{d}+p_{n}\omega_{m}\psi_{f}\;\\ J_{m}\frac{d\omega_{m}}{dt}=\frac{3}{2}p_{n}\psi_{f}i_{q}-T_{L}\;\;\;\;\;\;\;\;\;\;\;\;\;\;\;\;\;\;\;\;\;\;\;\;\;\;\;\;\;\;\;\;\;\;\;\;\;\;\;\;\;\;\;\;\;\;\;\;\;\;\;\;\;\;\;\;\;\;\;\;\;\;\;\; \end{array}\right.$$

Because it is a surface-mounted PMSM, Equation (32) can be transformed into the following form.

(33) $$\left\{\begin{array}{c} \frac{di_{q}}{dt}=\frac{1}{L_{q}}\left(-R_{s}i_{q}-p_{n}\omega_{m}\psi_{f}+u_{q}\right)\;\;\;\\ \frac{d\omega_{m}}{dt}=\frac{1}{J_{m}}\left(-T_{L}+\frac{3}{2}p_{n}\psi_{f}i_{q}\right)\;\;\;\;\;\;\;\;\;\;\;\;\;\;\;\;\; \end{array}\right.$$

The state variables are selected as follows [32].

(34) $$\left\{\begin{array}{c} x_{1}=\omega_{ref}-\omega_{m}\\ x_{2}={\dot{x}}_{1}=-{\dot{\omega }}_{m} \end{array}\right.$$

where *ω_ref_* is the reference speed of the PMSM.

Equation (33) is substituted into Equation (34), and the formula can be rewritten as follows:

(35) $$\left[\begin{array}{c} {\dot{x}}_{1}\\ {\dot{x}}_{2} \end{array}\right]=\left[\begin{array}{cc} 0 & 1\\ 0 & 0 \end{array}\right]\left[\begin{array}{c} x_{1}\\ x_{2} \end{array}\right]+\left[\begin{array}{c} 0\\ -\frac{3p_{n}\psi_{f}}{2J_{m}} \end{array}\right]{\dot{i}}_{q}$$

The non-singular SMS can be defined as follows [25,29]:

(36) $$s_{c}=x_{1}+\frac{1}{\lambda }x_{2}^{p/q}$$

where *λ* is a positive proportionality coefficient.

Taking the derivative of the non-singular SMS *s_c_* with respect to time, and the IFPRL shown in Equation (10) is substituted into it, we obtain the following expression:

(37) $$\dot{s}={\dot{x}}_{1}+\frac{p}{q\lambda }x_{2}^{p/q-1}\cdot {\dot{x}}_{2}=x_{2}+\frac{p}{q\lambda }x_{2}^{p/q-1}\cdot {\dot{x}}_{2}=slaw$$

The expression of the speed controller is shown as follows:

(38) $${\dot{i}}_{q}=\frac{2J_{m}}{3p_{n}\psi_{f}}\left(\lambda \frac{q}{p}{x_{2}}^{2-p/q}+k_{1}{\left|s_{c}/c\right|}^{a}\mathrm{sgn}(s_{c})+k_{2}{\left|s_{c}/c\right|}^{b\cdot \mathrm{sgn}(\left|s_{c}/c\right|-1)}s_{c}\right)$$

Thus, the reference current of the *q*-axis is expressed as follows:

(39) $$i_{q}=\frac{2J_{m}}{3p_{n}\psi_{f}}\int_{0}^{t}\left(\lambda \frac{q}{p}{x_{2}}^{2-p/q}+k_{1}{\left|s_{c}/c\right|}^{a}\mathrm{sgn}(s_{c})+k_{2}{\left|s_{c}/c\right|}^{b\cdot \mathrm{sgn}(\left|s_{c}/c\right|-1)}s_{c}\right)dt$$

### 5.2. Stability Analysis of NTSMC

In order to analyze the stability of the NTSMC, the following Lyapunov function is considered:

(40) $$V_{c}=\frac{1}{2}s_{c}^{2}$$

Differentiating *V_c_* with respect to time, this shows as follows:

(41) $$\begin{array}{l} \;\;\;\;\;\;{\dot{V}}_{c}=s_{c}{\dot{s}}_{c}\\ \;\;\;\;\;\;\;\;\;\;\;=s_{c}(x_{2}+\frac{p}{q\lambda }x_{2}^{p/q-1}\cdot {\dot{x}}_{2})\\ \;\;\;\;\;\;\;\;\;\;\;=s_{c}\left[\frac{p}{q\lambda }x_{2}^{p/q-1}\left(-k_{1}{\left|s_{c}/c\right|}^{a}\mathrm{sgn}(s_{c})-k_{2}{\left|s_{c}/c\right|}^{b\cdot \mathrm{sgn}(\left|s_{c}/c\right|-1)}s_{c}\right)\right]\\ \;\;\;\;\;\;\;\;\;\;\;=\frac{p}{q\lambda }x_{2}^{p/q-1}\left(-ck_{1}{\left|s_{c}/c\right|}^{1+a}-k_{2}{\left|s_{c}/c\right|}^{b\cdot \mathrm{sgn}(\left|s_{c}/c\right|-1)}s_{c}^{2}\right) \end{array}$$

Due to *c* > 0, *k*_1_ > 0, *k*_2_ > 0, and *p* and *q* (*p* > *q*) being positive odd numbers, Equation (41) can be simplified as:

(42) $${\dot{V}}_{c}=\frac{p}{q\lambda }x_{2}^{p/q-1}\left(-ck_{1}{\left|s_{c}/c\right|}^{1+a}-k_{2}{\left|s_{c}/c\right|}^{b\cdot \mathrm{sgn}(\left|s_{c}/c\right|-1)}s_{c}^{2}\right)\;\leq 0$$

Therefore, the designed NTSMC is asymptotically stable.

### 5.3. Design of Sensorless Speed Controller

**Figure 7:**
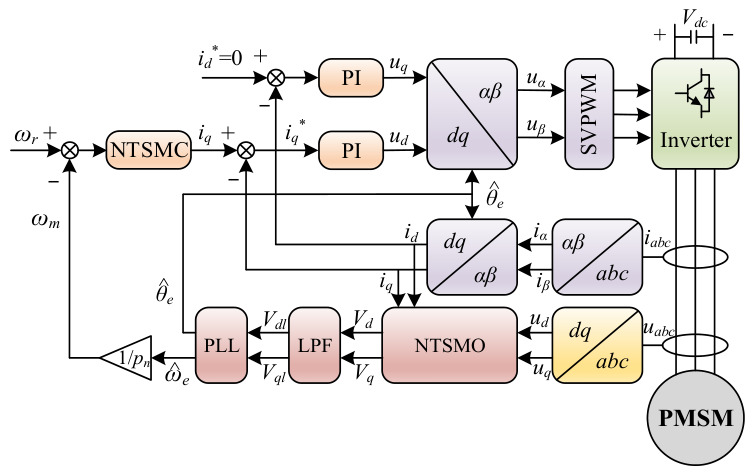
Schematic diagram of sensorless speed control for the PMSM drive system.

Finally, by combining the designed NTSMO, PLL, and NTSMC, the sensorless speed control of the PMSM drive system can be achieved, as shown in Figure 7.

## 6. Simulations and Experiments

### 6.1. Simulation and Experimental Conditions

**Figure 8:**
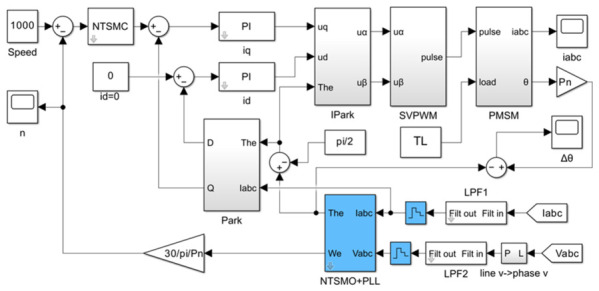
Simulation model of sensorless speed control for the PMSM.

**Figure 9:**
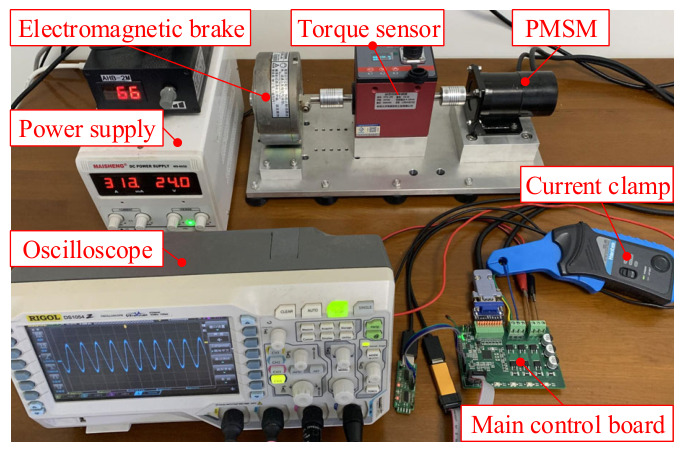
Experimental apparatus of sensorless speed control for the PMSM.

**Figure 10:**
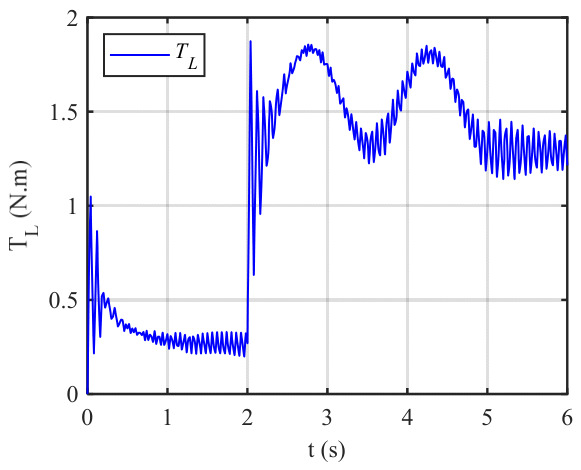
Load of the PMSM in simulations and experiments.

In order to verify the rotor position estimation and sensorless speed control method for the PMSM proposed in this paper, we constructed a simulation model using MATLAB/Simulink (as shown in Figure 8) and built a speed control experimental system (as shown in Figure 9). In the numerical simulation and experimental process, we used the scaled load characteristics in ref. [29] (as shown in Figure 10) to load the PMSM. The parameters of the PMSM are as follows: rated speed is 1000 r/min, stator resistance is 0.04 Ω, flux linkage is 0.0247 Wb, d-axis inductance is 0.438 mH, q-axis inductance is 0.438 mH, count of pole pairs is 2, and moment of inertia is 0.0008 kg.m^2^.

### 6.2. Simulation Results

#### 6.2.1. Rotor Speed and Position Estimation

**Figure 11:**
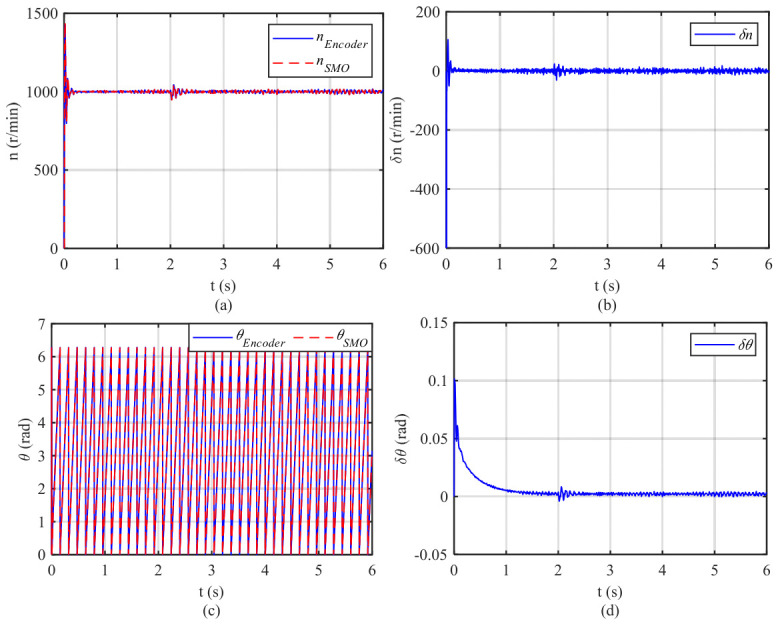
Simulation results of rotor speed and position estimation based on traditional SMO and PLL: (**a**) rotor speeds; (**b**) estimation error of rotor speeds; (**c**) rotor positions; (**d**) estimation error of rotor positions.

Figure 11 shows the simulation results of the rotor speed and position estimation for a PMSM based on the traditional SMO [8]. In Figure 11a, the blue solid line is the rotor speed of the PMSM detected by the mechanical position sensor, and the red dashed line is the estimated rotor speed of the PMSM based on traditional SMO [8]. The estimation error of rotor speed is shown in Figure 11b. It can be seen that there will be significant errors in the initial stage of rotor speed estimation, and when the load suddenly increases, it will cause the estimation error to increase. The estimated rotor position is shown in Figure 11c. The estimation error of rotor position is shown in Figure 11d. The estimation error of the rotor position gradually decreases, but the convergence speed is slow. This is because the performance limitations of the reaching law in traditional SMO [8] affect the performance of the final rotor position estimation. In addition, changes in load can also lead to an increase in the estimation error of the motor rotor position.

**Figure 12:**
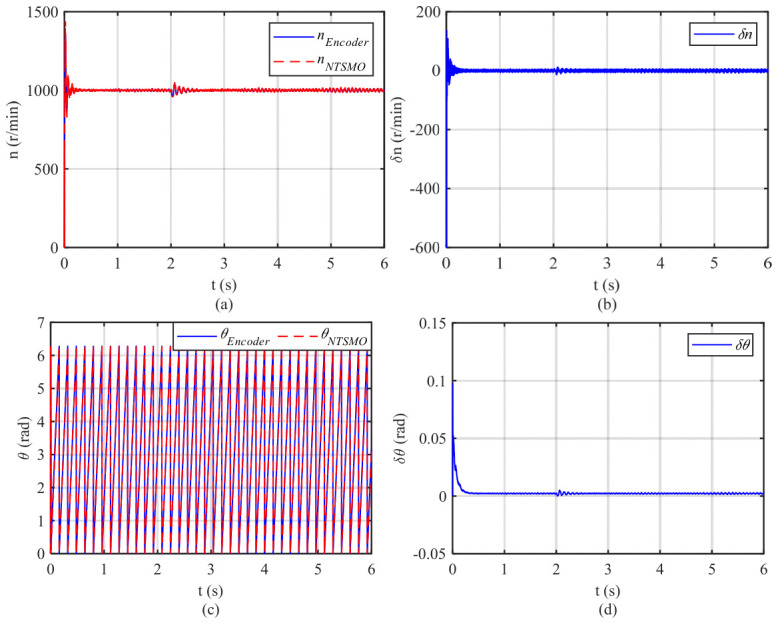
Simulation results of rotor speed and position estimation based on the proposed NTSMO and PLL: (**a**) rotor speeds; (**b**) estimation error of rotor speed; (**c**) rotor positions; (**d**) estimation error of rotor positions.

Similarly, the simulation results of the rotor speed and position estimation for a PMSM based on the proposed IFPRL-based NTSMO are shown in Figure 12. In Figure 12a, the blue solid line is the rotor speed of the PMSM detected by the mechanical position sensor, and the red dashed line is the estimated rotor speed of the PMSM based on the proposed IFPRL-based NTSMO. It can be seen that the two curves are basically consistent, which proves that the method proposed in this paper has successfully achieved the rotor speed estimation of the MSM. Compared to Figure 11b, the estimation error of rotor speed shown in Figure 12b is smaller. The estimated rotor position is shown in Figure 12c. Compared with Figure 11d, the convergence speed of the rotor position estimation error shown in Figure 12d is faster, and the error is smaller. This underscores the effectiveness of the IFPRL proposed in this paper, demonstrating improved reaching speed and overall performance. Furthermore, the proposed method has stronger robustness and anti-disturbance ability, particularly under varying load conditions.

In order to better compare the estimation effects of speed and rotor position between the two methods (traditional SMO [8] and proposed IFPRL-based NTSMO), we conducted a quantitative statistical analysis of the estimation curves in Figure 11 and Figure 12. The root mean square error (RMSE) of rotor speed estimation using the proposed method decreases from 19.97 r/min to 19.13 r/min, an improvement of 4.2%; the RMSE of rotor position estimation decreases from 0.0113 rad to 0.0049 rad, an improvement of 56.6%.

**Figure 13:**
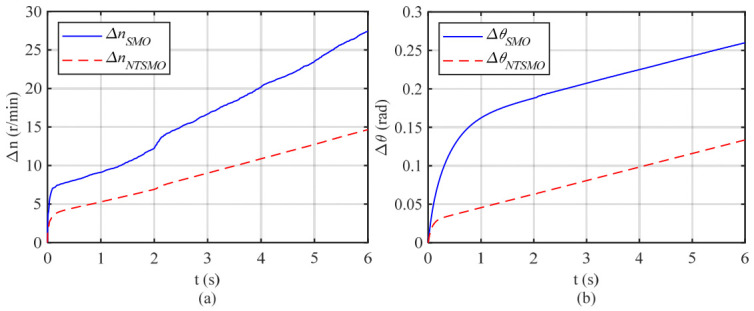
Simulation results of cumulative estimation errors of the traditional SMO method and proposed IFPRL-based NTSMO method: (**a**) cumulative rotor speed error Δ*n*; (**b**) cumulative rotor position error Δ*θ*.

In addition, we calculated the cumulative estimation errors, as shown in Figure 13. The comparison reveals that the proposed NTSMO incorporating FPRL achieves significantly smaller cumulative errors in both rotor speed and position estimation. Specifically, the cumulative absolute error of rotor speed estimation decreases from 27.43 r/min to 14.66 r/min, an improvement of 46.5%; the cumulative absolute error of rotor position estimation decreases from 0.26 rad to 0.13 rad, an improvement of 50.0%. The above quantitative analysis results demonstrate that the proposed method can track the actual rotor speed and position signals of the PMSM more quickly and accurately, providing stable and reliable state feedback for high-performance sensorless speed control.

#### 6.2.2. Sensorless Speed Control

**Figure 14:**
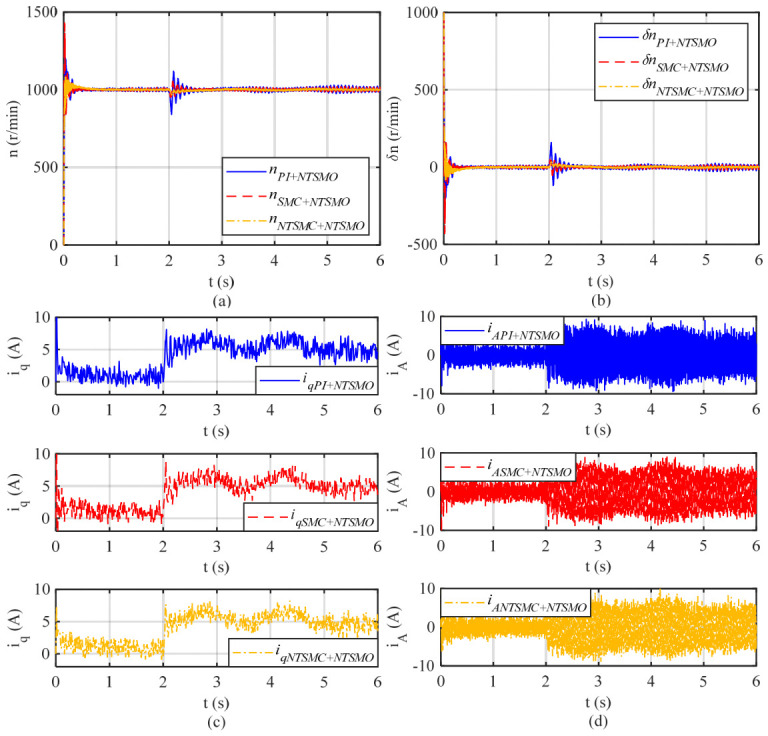
Simulation results of sensorless speed control for the PMSM: (**a**) rotor speeds; (**b**) control errors of rotor speeds; (**c**) *q*-axis currents; (**d**) A-phase currents.

The aforementioned analysis demonstrates the effectiveness of the IFPRL-based NTSMO method proposed in this paper, which we subsequently implemented for sensorless speed control of a PMSM drive system. Meanwhile, the PI [30] and SMC [2] methods were compared with the IFPRL-based NTSMC method proposed in this paper, and the results are shown in Figure 14. In Figure 14a, the blue solid line represents the results based on the PI [29] speed controller and proposed IFPRL-based NTSMO, the red dashed line represents the results based on the SMC speed controller [2] and proposed IFPRL-based NTSMO, and the yellow dotted line represents the results based on the proposed IFPRL-based NTSMC + NTSMO method. The corresponding speed control error curves of the PMSM are shown in Figure 14b. It can be seen that due to the initial rotor position estimation error, all three methods have significant overshoot. However, compared with PI [29] and SMC [2] speed controllers, the IFPRL-based NTSMC method proposed in this paper has the smallest error and better anti-disturbance when the load changes (the load of the PMSM is shown in Figure 10). Figure 14c,d show the *q*-axis currents and A-phase currents of the PMSM corresponding to the three methods, respectively.

In order to better compare the sensorless speed control effects of three methods (PI [29] + NTSMO, SMC [2] + NTSMO, and NTSMC + NTSMO) on the PMSM drive system, we conducted a quantitative statistical analysis of the motor speed control results in Figure 14. In terms of the peak speed indicator, the peak speed of the proposed NTSMC + NTSMO method is 1092.50 r/min, which is 9.3% lower than that of the PI + NTSMO method and 23.8% lower than that of the SMC + NTSMO method, indicating that this method can effectively suppress system overshoot and significantly improve dynamic response performance. Regarding the RMSE of speed control, the NTSMC + NTSMO method achieves only 32.92 r/min, representing a reduction of 14.6% compared to the PI + NTSMO method and 14.1% compared to the SMC + NTSMO method.

**Figure 15:**
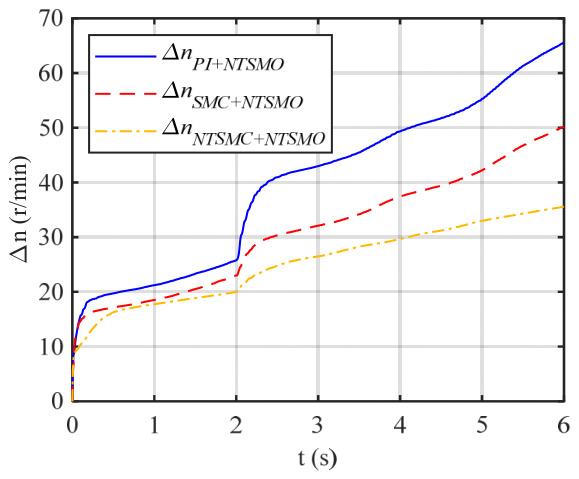
Simulation results of the cumulative sensorless control errors of different sensorless speed control methods.

Furthermore, we additionally calculated the cumulative absolute error of speed control for the three methods, as shown in Figure 15. The comparison results show that the proposed sensorless speed control method yields the smallest cumulative absolute error, only 35.56 r/min, which is 45.8% lower than the PI + NTSMO method and 29.2% lower than the SMC + NTSMO method, demonstrating that this method possesses the best overall tracking accuracy throughout the entire control process. The above quantitative analysis results fully prove that the proposed sensorless speed control method can track the actual speed signal of the PMSM more rapidly and accurately, providing more stable and reliable control performance for the system.

### 6.3. Experiment Results

**Figure 16:**
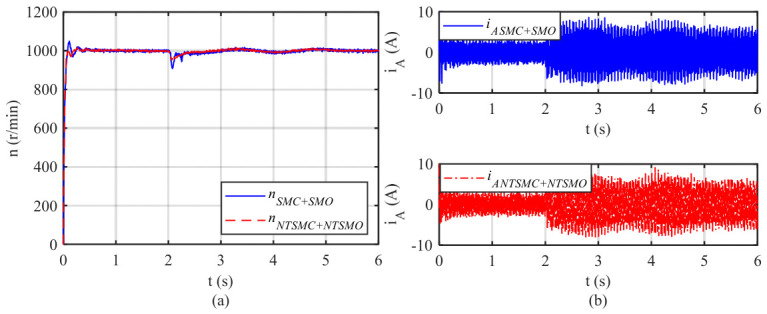
Experimental results of sensorless speed control for the PMSM: (**a**) rotor speeds; (**b**) A-phase currents.

Using the experimental platform shown in Figure 9, the sensorless speed control strategy for the PMSM proposed in this paper was validated, and the results are shown in Figure 16. In Figure 16a, the blue solid line represents the results based on traditional SMC [2] and SMO [8], while the red dashed line represents the results based on the proposed IFPRL-based NTSMC + NTSMO method. It can be seen that compared to the traditional method, the proposed sensorless speed control method for the PMSM has smaller overshoot. Meanwhile, the method proposed in this paper has stronger speed control stability and anti-disturbance under load changes (the load of the PMSM is shown in Figure 10). Figure 16b shows the A-phase current of the PMSM corresponding to the two methods.

**Figure 17:**
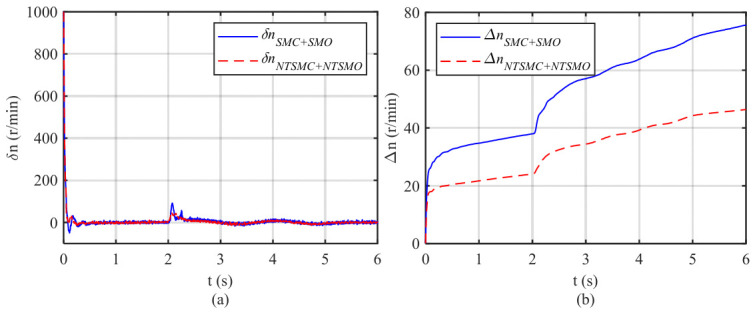
Experimental results of the cumulative sensorless control errors for the PMSM: (**a**) control errors of rotor speeds; (**b**) cumulative errors of rotor speeds.

In order to better compare the effectiveness of two sensorless speed control methods (SMC [2] + SMO [8] and NTSMC + NTSMO) for the PMSM, we calculated the speed control errors of the PMSM, as shown in Figure 17a, and the corresponding cumulative speed control errors are shown in Figure 17b. Furthermore, we performed a quantitative statistical analysis on these experimental data. In terms of the peak speed indicator, the peak speed of the proposed IFPRL-based NTSMC + NTSMO method is 1017.15 r/min, which is 3.2% lower than that of the traditional SMC + SMO method, indicating that this method can still effectively suppress system overshoot under actual operating conditions. Regarding the RMSE of speed control, the IFPRL-based NTSMC + NTSMO method achieves 46.22 r/min, representing a reduction of 2.7% compared to the traditional SMC + SMO method. Most notably, in terms of the cumulative absolute error, the proposed method is only 46.38 r/min, which is substantially reduced by 38.7% compared to the conventional SMC + SMO method, demonstrating the best overall tracking accuracy. The above quantitative analysis results show that the proposed sensorless speed control method significantly outperforms the traditional method across all performance indicators. The experimental results are highly consistent with the performance trends observed in the simulation, fully validate the effectiveness and robustness of the proposed method in practical applications, and can provide more stable and reliable control performance for the PMSM drive system.

## 7. Conclusions

In this paper, a novel sensorless speed control strategy for PMSM drive systems, which integrates the NTSMO and NTSMC with the proposed IFPRL, was presented. The IFPRL is developed to simultaneously improve the response speed, suppress sliding mode chattering, and enhance the anti-disturbance performance of the closed-loop system. Finally, the effectiveness and superiority of the proposed method are verified via extensive numerical simulations and hardware experiments.

By establishing the second-order simulation model with disturbances, it has been proven that, compared with the ERL and NSMRL reported in the existing literature, the proposed IFPRL achieves a faster reaching speed and more effective suppression of inherent chattering. Subsequently, under typical PMSM load characteristics, the performance of the IFPRL-based NTSMO is evaluated against that of the traditional SMO. Simulation results show that the proposed observer reduces the RMSE of rotor position estimation by 56.6% (from 0.0113 rad to 0.0049 rad) and the cumulative absolute error by 50.0%. For rotor speed estimation, it decreases the RMSE by 4.2% and the cumulative absolute error by 46.5%, validating its superior state estimation capability for sensorless control applications. In terms of speed closed-loop control, the IFPRL-based NTSMC is compared with traditional PI and SMC controllers. Simulation results indicate that the proposed controller achieves a 14.6% reduction in speed tracking RMSE relative to the PI controller and a 14.1% reduction relative to the SMC controller, with the cumulative absolute error of speed control decreased by 45.8% and 29.2%, respectively. Notably, it exhibits significantly stronger anti-interference capability under sudden load variations. The hardware experimental results are highly consistent with the simulation findings. Compared with the traditional SMC + SMO scheme, the proposed IFPRL-based NTSMC + NTSMO method reduces the cumulative absolute error of speed control by 38.7%, with a 2.7% reduction in speed control RMSE. This consistency further confirms the feasibility and practicality of the proposed method under real-world operating conditions.

For future work, we will focus on application research under complex working conditions, specifically addressing the practical challenges in real drive systems, such as controller saturation, signal time delay, and other non-ideal characteristics, to further enhance the adaptability and reliability of the control system in extreme operating environments.

## Data Availability

Data available on request from the authors.
